# Higher throughput drug screening for rare respiratory diseases: readthrough therapy in primary ciliary dyskinesia

**DOI:** 10.1183/13993003.00455-2020

**Published:** 2021-10-14

**Authors:** Dani Do Hyang Lee, Daniela Cardinale, Ersilia Nigro, Colin R. Butler, Andrew Rutman, Mahmoud R. Fassad, Robert A. Hirst, Dale Moulding, Alexander Agrotis, Elisabeth Forsythe, Daniel Peckham, Evie Robson, Claire M. Smith, Satyanarayana Somavarapu, Philip L. Beales, Stephen L. Hart, Sam M. Janes, Hannah M. Mitchison, Robin Ketteler, Robert E. Hynds, Christopher O'Callaghan

**Affiliations:** 1 UCL Great Ormond Street Institute of Child Health, London, UK; 2 Lungs for Living Research Centre, UCL Respiratory, Division of Medicine, University College London, London, UK; 3 Centre for PCD Diagnosis and Research, Dept of Respiratory Sciences, University of Leicester, Leicester, UK; 4 Ciliary Disease Section, Genetics and Genomic Medicine Research and Teaching Dept, UCL Great Ormond Street Institute of Child Health, London, UK; 5 Dept of Human Genetics, Medical Research Institute, Alexandria University, Alexandria, Egypt; 6 Developmental Biology and Cancer, UCL Great Ormond Street Institute of Child Health, London, UK; 7 MRC Laboratory for Molecular Cell Biology, University College London, London, UK; 8 Leeds Institute for Medical Research, University of Leeds, Leeds, UK; 9 Dept of Pharmaceutics, UCL School of Pharmacy, University College London, London, UK; 10 UCL Cancer Institute, University College London, London, UK; 11 D.D.H. Lee and D. Cardinale contributed equally; 12 R.E. Hynds and C. O'Callaghan contributed equally to this article as lead authors and supervised the work

## Abstract

**Background:**

Development of therapeutic approaches for rare respiratory diseases is hampered by the lack of systems that allow medium-to-high-throughput screening of fully differentiated respiratory epithelium from affected patients. This is a particular problem for primary ciliary dyskinesia (PCD), a rare genetic disease caused by mutations in genes that adversely affect ciliary movement and consequently mucociliary transport. Primary cell culture of basal epithelial cells from nasal brush biopsies followed by ciliated differentiation at the air–liquid interface (ALI) has proven to be a useful tool in PCD diagnostics but the technique's broader utility, including in pre-clinical PCD research, has been restricted by the limited number of basal cells that can be expanded from such biopsies.

**Methods:**

We describe an immunofluorescence screening method, enabled by extensive expansion of basal cells from PCD patients and the directed differentiation of these cells into ciliated epithelium in miniaturised 96-well transwell format ALI cultures. As proof-of-principle, we performed a personalised investigation in a patient with a rare and severe form of PCD (reduced generation of motile cilia), in this case caused by a homozygous nonsense mutation in the *MCIDAS* gene.

**Results:**

Initial analyses of ciliary ultrastructure, beat pattern and beat frequency in the 96-well transwell format ALI cultures indicate that a range of different PCD defects can be retained in these cultures. The screening system in our proof-of-principal investigation allowed drugs that induce translational readthrough to be evaluated alone or in combination with nonsense-mediated decay inhibitors. We observed restoration of basal body formation but not the generation of cilia in the patient's nasal epithelial cells *in vitro.*

**Conclusion:**

Our study provides a platform for higher throughput analyses of airway epithelia that is applicable in a range of settings and suggests novel avenues for drug evaluation and development in PCD caused by nonsense mutations.

## Abstract 2

**Primary cell culture of nasal epithelial cells (including differentiation to multiciliated cells) from patients with primary ciliary dyskinesia enabled immunofluorescence-based screening in miniaturised air–liquid interface cultures** https://bit.ly/3rjoxBF

## Introduction

Primary ciliary dyskinesia (PCD) is a rare autosomal recessive genetic disorder arising from abnormalities in the structure and function of motile cilia. The disease is characterised by recurrent respiratory tract infections and early onset bronchiectasis, chronic nasal symptoms and sinusitis, and, in many patients, a significant hearing deficit due to glue ear. Genetically, PCD is a heterogeneous disorder, with variants in more than 40 different genes accounting for disease in ∼70% of PCD patients [1–3]. While the specific ultrastructural defects caused by these mutations vary, they generally occur in genes that encode proteins involved in axonemal structure, regulatory complexes, ciliary assembly or ciliary transport [4]. Abnormalities in ciliary function depend on the underlying defect, which may result in static cilia, cilia with a markedly reduced beat amplitude, cilia that rotate or cilia that are hyperkinetic.

As a result of disease diversity, there is no single PCD diagnostic test: clinical history, genetic analyses, low nasal nitric oxide levels and direct observation by light microscopy of ciliated epithelial cells in nasal brush biopsies using high-speed video analysis as well as analyses of ciliary fine structure by electron microscopy are used, often in combination, to reach a PCD diagnosis [5]. In specialised centres, cell culture is used to improve the diagnosis because ciliary dyskinesia secondary to infection and inflammation can make interpretation difficult. Examining cultured cells can also reduce the requirement for re-biopsy when biopsy samples do not contain ciliated cells [6], and cell culture is advantageous when characterising atypical PCD phenotypes such as inner dynein arm defects, ciliary disorientation or reduced generation of multiple motile cilia.

The diversity of patient genotypes and phenotypes in PCD suggest it as a fertile ground for a personalised medicine approach. However, nasal epithelial cell cultures from brush biopsies of PCD patients have been of limited value because basal cells undergo senescence soon after culture initiation. Because few cells are obtained during nasal or lower airway biopsy procedures, the majority of investigations to date seed cells directly in air–liquid interface (ALI) cultures (in which cells are cultured in transwells and fed basally in order to expose them to air) or after minimal passaging [7]. Improvements in epithelial cell culture techniques have led to methods that allow extensive expansion of airway [8] and nasal [9] epithelial cells from healthy donors and patients with genetic diseases such as cystic fibrosis [10–12]. In PCD, a previous study of *RSPH1*-mutant PCD patients indicated that patient phenotypes are recapitulated upon differentiation *in vitro* [13]. Here, we demonstrate extensive expansion of basal cells from PCD patients with diverse causative mutations in 3T3-J2 fibroblast feeder cell co-culture in medium containing Y-27632, a Rho-associated protein kinase (ROCK) inhibitor, and the maintenance of the patients’ ciliary phenotypes in ALI cultures. We also miniaturise ALI cultures from PCD patients and healthy volunteers to a 96-well format that allows higher throughput screening than conventional ALI culture. Around a quarter of PCD patients carry nonsense mutations that introduce a premature termination codon (PTC) into the mRNA, leading to an absence of the protein or the production of inactive, truncated forms [14]. PTC-“readthrough” can bypass the PTC, leading to partial or full expression of functional protein [15]. Several readthrough agents have been described, including the antibiotic gentamicin and ataluren, a drug in development for Duchenne muscular dystrophy [16] that has also been trialled in cystic fibrosis [17]. Analysis of PCD PTCs cloned into luciferase reporter constructs in HEK293FT cells suggested that a subset of PTCs could be suppressed by aminoglycoside readthrough agents [18].

Focusing on one of our well-characterised patients with a reduced generation of multiple motile cilia (RGMC) ciliopathy caused by a mutation in the multiciliate differentiation and DNA synthesis associated cell cycle gene (*MCIDAS*) (c.441C>A; p.Cys147*) [19], we used an immunofluorescence screening approach to assess the formation of multiple basal bodies, structures that organise microtubules during multi-ciliogenesis, in miniaturised ALI cultures. Despite the observation of isolated basal bodies in nasal brushing from patients with this condition, the formation of multiple basal bodies is compromised by the lack of this protein in ALI cultures [19, 20]. Combinations of readthrough agents and inhibitors of nonsense-mediated decay (NMD) were able to restore the formation of basal bodies, but not cilia, in the patient's epithelial cells.

## Materials and methods

### Subjects and genetic analysis

Affected individuals were diagnosed with PCD using standard diagnostic screening by the national commissioning group-funded PCD diagnostic service, according to European Respiratory Society diagnostic guidelines [5]. Samples for genetic screening were collected under ethical approval obtained through the London Bloomsbury Research Ethics Committee (08/H0713/82). Informed written consent was obtained from all participants or their guardians prior to enrolment in the study. Genetic screening was performed as previously described [21] or using a targeted next-generation sequencing panel approach [22].

### Isolation of epithelial cells from nasal brush biopsies

Human nasal epithelial cell cultures were derived from nasal brush biopsies taken with informed consent. Ethical approval was obtained through the National Research Ethics Committee (REC reference 14/NW/0128) and UCL Research Ethics (reference 4735/001). Biopsies were received on ice in transport medium which consisted of Medium 199 (Life Technologies) supplemented with 100 U·mL^−1^ penicillin, 100 µg·mL^−1^ streptomycin (Gibco), 25 µg·mL^−1^ amphotericin B (Gibco) and 20.5 µg·mL^−1^ sodium deoxycholate (Gibco). Cells were plated directly into T25 flasks in bronchial epithelial growth medium (“P0”; BEGM, Lonza) and cultured for 7–10 days prior to first passage. After the first passage, cells were either expanded in BEGM or in co-culture with 3T3-J2 fibroblasts as described below.

### Feeder cell culture

3T3-J2 mouse embryonic fibroblasts were grown in DMEM (Gibco) supplemented with 100 U·mL^−1^ penicillin, 100 µg·mL^−1^ streptomycin (Gibco) and 8% bovine serum (Gibco). Fibroblasts were cultured at 37°C with 5% CO_2_ and medium was changed three times per week. To generate feeder layers, cells were mitotically inactivated by treatment with 4 µg·mL^−1^ mitomycin C (Sigma) in culture medium for 2–3 h. Inactivated fibroblasts were then trypsinised and plated at a density of 20 000 cells·cm^−2^ in fibroblast growth medium and allowed to adhere overnight.

### 3T3-J2 co-culture of human nasal epithelial cells

Co-culture with 3T3-J2 fibroblasts was performed as previously described [23]. Epithelial cell culture medium consisted of DMEM (Gibco) and F12 (Gibco) in a 3:1 ratio with 1X penicillin/streptomycin (Gibco) and 5% FBS (Gibco) supplemented with 5 μM Y-27632 (Cambridge Bioscience), 25 ng·mL^−1^ hydrocortisone (Sigma), 0.125 ng·mL^−1^ EGF (Sino Biological), 5 μg·mL^−1^ insulin (Sigma), 0.1 nM cholera toxin (Sigma), 250 ng·mL^−1^ amphotericin B (Fisher Scientific) and 10 μg·mL^−1^ gentamicin (Gibco). Streptomycin and gentamicin were removed from the medium for RGMC cell culture. Epithelial cells were cultured at 37°C and 5% CO_2_ with three changes of medium per week. Population doublings (PD) were calculated as PD=3.32×(log(cells harvested/cells seeded)). Cryopreservation was performed using Profreeze freezing medium (Lonza) according to the manufacturer's instructions and using epithelial cell culture medium as defined above to dilute the stock solution.

### Differentiation in ALI cultures

At confluence, basal cells were separated from feeder cells using differential trypsinisation [8], in which a brief initial incubation with 0.05% trypsin/EDTA was sufficient to remove fibroblasts but epithelial cells remained adherent. After washing, a second, longer incubation efficiently removed epithelial cells. Basal cells were seeded on collagen I-coated, semi-permeable membrane supports (Transwell-Col, 0.4 µm pore size; Corning) in submerged culture in BEGM containing 5 µM Y-27632. For 12-well transwells, 1×10^6^ cells were seeded per membrane in 250 µL medium. Miniaturised ALI cultures were performed in 96-transwell plates (Corning; 1 µM pores, polyester membrane) where 0.2×10^6^ cells were seeded per membrane in 75 µL medium. Upon confluence, cells were fed only from the basolateral side with ALI medium. ALI medium consisted of 50% BEGM and 50% hi-glucose minimal essential medium (DMEM, Gibco) containing 100 nM additional retinoic acid (Sigma Aldrich). For 96-transwell ALI cultures, BEGM was replaced with Promocell's Airway Epithelial Cell Growth Medium. Medium was exchanged three times per week and gentle washing with bronchial epithelial basal medium (BEBM) or Promocell basal medium removed the mucus produced on the apical surface once per week. Basal cell passage numbers for each experiment are indicated in supplementary table S1.

### Immunofluorescence

Cultured basal cells were seeded in 8-well chamber slides or fixed directly on the transwell membrane by incubation in 4% paraformaldehyde for 30 min at room temperature. Cells were stored at 4**°**C in PBS until the time of staining. Cells were blocked and permeabilised using block buffer (3% BSA in PBS containing 0.01% Triton X-100) at room temperature for 1 h, prior to overnight staining with primary antibody (in 1% BSA in PBS) at 4°C. Primary antibodies used were anti-C-Nap1 (Sigma; 1:50) for basal body staining, anti-β-tubulin (Abcam; 1:100) and anti-MUC5AC (Invitrogen; 1:100). Cells were washed three times in PBS for 5 min and secondary antibody (in 1% BSA in PBS; Molecular Probes; AlexaFluor dyes) was applied for 2 h at room temperature. Hoechst 33258 staining solution (Sigma) was applied for 20 min at room temperature as a nuclear counterstain prior to imaging.

### Immunofluorescence-based screening in miniaturised ALI cultures

Cells from the patient examined in the screening assays were isolated and expanded in 3T3+Y cell culture conditions as described above. Gentamicin (50 and 100 μg·mL^−1^) and ataluren (5 and 10 μg·mL^−1^) alone or in combination with amlexanox (1.5 µg·mL^−1^) and escin (5.6 and 11.3 µg·mL^−1^) were applied to ALI cultures basolaterally from the onset of differentiation (*i.e.* at air-lift). Drugs were refreshed each time the cultures were fed. Cells were fixed for immunofluorescence at day 12 post-ALI to assess basal body formation. Selected drugs and drug/combinations from the screening were also applied to 12-well transwell ALI cultures and cells were collected for Western blot and quantitative PCR (qPCR) analysis after 7 days.

To assess basal body formation, cells were imaged directly in 96-well transwells using automated confocal microscopy (Opera Phenix High-Content Screening System, PerkinElmer, ×5 objective). For higher magnification imaging, cells were mounted in 80% glycerol, 3% n-propylgallate (in PBS) mounting medium and images were obtained using an inverted Zeiss LSM 710 confocal microscope. Analysis of basal bodies was performed using a custom ImageJ macro; the analysis approach is summarised in supplementary figure S1 and the macro is freely available at the following link: https://github.com/DaleMoulding/Fiji-Macros/blob/master/DanielaSpotClustersv9.ijm.

### Transepithelial electrical resistance

Transepithelial electrical resistance (TEER) values were measured using an EVOM2 resistance meter and Endohm chamber (World Precision Instruments) with a cup size appropriate for the size of the culture insert (6 mm culture cup for 24-well transwells and 12 mm culture cup for 12-well transwells). Transwells were placed into the culture cup and readings were taken after the TEER reading had stabilised (typically 5–10 s). Readings were taken from three independent transwells once the cultures were fully differentiated, *i.e.* after at least 5 weeks of ALI culture, to obtain an average TEER value for each culture (9–12 readings per donor).

### Ciliary beat frequency and beat pattern

For ciliary analyses, the ciliated epithelium was removed from the transwell insert by gentle scraping with a spatula. Cells were washed with transport medium (as described above) and dissociated by gentle pipetting. The cell suspension was divided to allow ciliary beat frequency and beat pattern analyses on unfixed cells and transmission electron microscopy (TEM) analysis on cells fixed in glutaraldehyde.

Beating cilia were recorded using a digital high-speed video camera (Motion Pro 4x; IDT) at a rate of 500 frames·s^−1^ using a ×100 objective [24]. Ten videos were acquired for each donor, each containing approximately 7–10 ciliated cells, and ciliary beat frequency of individual ciliated cells was determined by counting the number of frames required for five full sweeps of a clearly visible ciliary tip. This was converted to ciliary beat frequency, where ciliary beat frequency=500/(number frames for 5 beats)×5. The percentage of dyskinetic ciliated cells relative to the total number of motile ciliated cells was calculated [25]. To analyse ciliary activity in cells differentiated in 96-well transwell ALI cultures, nasal epithelial cells were observed using an inverted microscope system (Nikon Ti-U) with a ×20 objective. At least 20 top-down videos per donor were recorded and ciliary beat frequency was analysed using the ImageJ plugin CiliaFA [26].

### qPCR

For qPCR, cultured epithelial cells were collected and mRNA was extracted in TRIzol and stored at −80°C. Cultures containing 3T3-J2 fibroblasts were differentially trypsinised to remove feeder cells as described above. After thawing on ice, total RNA was isolated using a Direct-zol RNA Isolation Kit (Zymo Research). RNA quantity was assessed using a Nanodrop One system and reverse transcription was performed using qScript cDNA SuperMix (Quantabio). The following Taqman pre-designed, inventoried probes, along with 2X PCR Master Mix (Applied Biosciences), were used for qPCR reactions: *KRT5* (Hs00361185_m1), *KRT14* (Hs00265033_m1), *KRT8* (Hs01595539_g1), *TP63* (Hs00978339_m1), *NGFR* (Hs00609977_m1), *ITGA6* (Hs01041011_m1), *MUC5AC* (Hs01365616_m1), *MUC5B* (Hs00861595_m1), *FOXJ1* (Hs00230964_m1) and *B2M* (Hs00187842_m1). qPCR was performed under standard conditions using an Eppendorf Real-Time PCR machine. Relative RNA quantitation was based on ΔCT calculations and all samples were compared to the housekeeping gene β2-microglobulin. For *MCIDAS* detection, primers (supplementary table S2) and Power SYBR™ Green PCR Master Mix (Thermo Fisher Scientific) were used in standard conditions and relative RNA quantification was normalised to the housekeeping gene *GAPDH*.

### Western blot

Cells were collected from transwells by scraping with a spatula, then pelleted and washed twice with PBS. The nuclear protein fraction was extracted with NE-PER Nuclear and Cytoplasmic Extraction Reagents (Thermo Fisher Scientific) and the concentration determined with the Pierce BCA assay kit (Thermo Fisher Scientific). Nuclear extracts were fractionated by SDS-PAGE and transferred to a PVDF membrane (for 1 h at 100 V). Membranes were blocked in 5% (w/v) milk in PBST (3.2 mM Na_2_HPO_4_, 0.5 mM KH_2_PO_4_, 1.3 mM KCl, 135 mM NaCl, 0.05% Tween 20, pH 7.4) for 1 h at room temperature and then incubated at 4°C overnight with primary antibodies (anti-multicilin, Atlas Antibodies, 1:500; anti-HDAC1, NovusBio, 1:500). Membranes were then washed three times in PBST and incubated with horseradish peroxidase-conjugated anti-rabbit antibodies (Cell Signalling Technologies; 1:3000) for 1 h. Blots were washed with PBST three times and developed with an ECL system (Bio-Rad) according to the manufacturer's protocol.

### TEM

Epithelial cells were scraped from transwells into Medium 199 and fixed in 4% glutaraldehyde in Sorensen's phosphate buffer (pH 7.4). Cells were then re-suspended in Sorensen's buffer and stored at 4°C until they were further processed to resin as previously described [25]. Ultrathin (70 nm) sections were cut and collected on copper grids, stained in 1% uranyl acetate, counterstained in Reynold's lead phosphate and examined by TEM [27].

### Statistics

Statistical analyses were performed using GraphPad Prism using the statistical tests indicated in figure legends.

## Results

### Cell culture expansion of PCD patient basal epithelial cells

**FIGURE 1:**
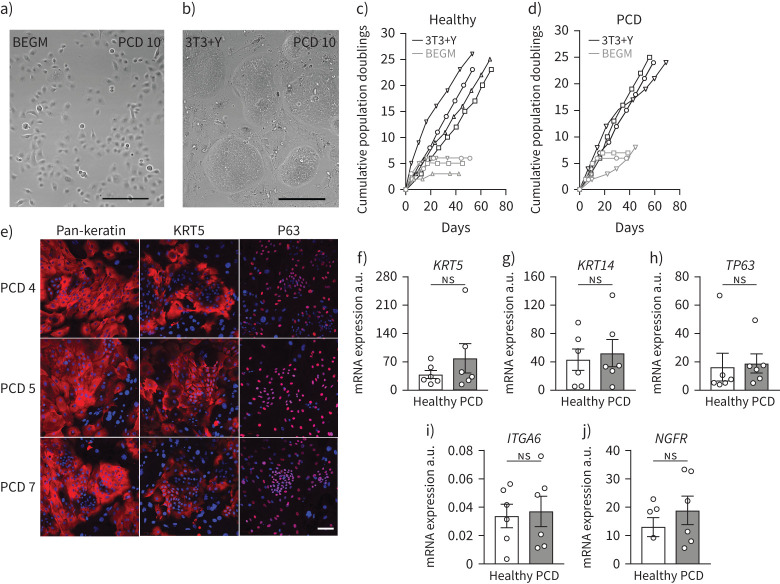
Expansion of nasal epithelial basal cells from patients with primary ciliary dyskinesia (PCD). a, b) Bright-field microscopy showing a representative example of nasal epithelial cells (PCD 10) in bronchial epithelial growth medium (BEGM) culture at passage 1 (a) and in 3T3+Y culture at passage 2 (b). Scale bar: 100 μm. c) Cumulative population doublings for four healthy donor nasal epithelial cell cultures in BEGM and 3T3+Y. d) Cumulative population doublings for three PCD patient nasal epithelial cells in BEGM and 3T3+Y. e) Representative examples of immunofluorescence images showing pan-keratin, keratin 5 (KRT5) and P63 expression (red) in three PCD patient 3T3+Y cultures. The nuclear counterstain DAPI is shown (blue). Scale bar: 100 µm. f–j) Quantitative PCR analysis comparing the expression of *KRT5* (f), *KRT14* (g), *TP63* (h), integrin α6 (*ITGA6*) (i) and nerve growth factor receptor (*NGFR*) (j) between healthy and PCD donor basal cell cultures in 3T3+Y. p-values were derived using two-tailed paired t-tests with significance set at p<0.05. a.u.: arbitrary units; ns: nonsignificant.

Brush biopsy-derived nasal epithelial cells were cultured from 12 PCD patients with a range of genetic defects, one patient with an RGMC ciliopathy and 11 healthy donors with no known ciliary defects (table 1). After isolation in BEGM [28, 29], cultures were expanded separately in both BEGM (figure 1a) and in co-culture with 3T3-J2 mouse embryonic fibroblast feeder cells in the presence of a ROCK inhibitor (Y-27632; 3T3+Y) (figure 1b). In BEGM, nasal epithelial cells senesced after three to four passages in both healthy (figure 1c) and PCD cultures (figure 1d). In contrast, and consistent with previous observations [9], expansion in 3T3+Y was rapid and was sustained for at least eight passages (figure 1c, d). 3T3+Y cultures from healthy and PCD donors were morphologically indistinguishable and, as expected, PCD cultures expressed the basal cell-associated proteins keratin 5 and P63 (figure 1e). Further, comparing the abundance of the basal cell-associated mRNAs *KRT5* (figure 1f), *KRT14* (figure 1g), *P63* (figure 1h), *ITGA6* (figure 1i) and *NGFR* (figure 1j) between control and PCD nasal cultures confirmed that cells in both control and PCD cultures are basal epithelial cells.

**TABLE 1:** Subject-specific CBP in biopsies and cell cultures with PCD

**Patient**	**CBP initial assessment**	**Ciliary defect (EM)**	**Genetic cause**	**CBP BEGM culture**	**CBP 3T3+Y culture**
**Healthy 1**		No known ciliary defects		Normal	Normal
**Healthy 2**		No known ciliary defects		Failed ALI	Normal (5% dyskinetic**^¶^**)
**Healthy 3**		No known ciliary defects		Normal (8% dyskinetic**^¶^**)	Normal
**Healthy 4**		No known ciliary defects		Normal (3% dyskinetic**^¶^**)	Normal (2% dyskinetic**^¶^**)
**Healthy 5**		No known ciliary defects		Not tested	Not tested
**Healthy 6**		No known ciliary defects		Not tested	Not tested
**Healthy 7**		No known ciliary defects		Not tested	Not tested
**Healthy 8**		No known ciliary defects		Not tested	Not tested
**Healthy 9**		No known ciliary defects		Not tested	Not tested
**Healthy 10**		No known ciliary defects		Not tested	Not tested
**Healthy 11**		No known ciliary defects		Not tested	Not tested
**PCD 1**	Static and flickering	Outer dynein arm	CCDC114 homozygous (p.Arg113*)	100% static	100% static
**PCD 2**	Stiff bottom/flexing top	Inner and outer dynein arm	CCDC65 homozygous (p.Glu220*)	1% static, 99% stiff	19% static, 81% stiff
**PCD 3** **^#^**	Static and flickering	MTD-L-IDA (radial spoke)	CCDC40 heterozygous (p.Glu66Argfs*)	69% static, 8% stiff, 23% twitch**^+^**	88% static, 2% stiff, 10% twitch**^+^**
**PCD 4**	Static and dyskinesia	Inner dynein arm, radial spoke, microtubular defect	CFAP300 compound heterozygous (p.Gln52*/p.Val63Asp)	99% static, 1% twitch**^+^**	100% static
**PCD 5**	Stiff^§^ and circular^ƒ^	Microtubular defect, transposition	RSPH4A homozygous (p.Gln154*)	9% static^§^, 91% stiff^§^, circular^ƒ^	4% static^§^, 96% stiff^§^, circular^ƒ^
**PCD 6**	Static and stiff	Inner dynein arm	CCDC40 homozygous (p.Ala83Valfs*84)	Failed ALI	19% static, 79% stiff, 2% twitch**^+^**
**PCD 7^#^**	Static and stiff	Inner dynein arm	CCDC103 heterozygous (p.His154Pro)	3% static, 97% stiff	6% static, 94% stiff
**PCD 8**	Static and twitching	Inner and outer dynein arm	RSPH4A homozygous (p.Gln154*)	100% static	100% static
**PCD 9**	Static	Outer dynein arm	DNAI2 homozygous (p.Arg247*)	Failed ALI	100% static
**PCD 10**	Static	Outer dynein arm	CFAP300 homozygous (p.Arg33Glyfs*44)	Failed ALI	100% static
**PCD 11**	Static and twitching	MTD-L-IDA (Radial spoke)	CCDC39 homozygous (p.Glu655Glyfs*23)	Not tested	Not tested
**PCD 12**	Circular^ƒ^	Transposition	RSPH4A homozygous (p.Trp24*)	Not tested	Not tested
**RGMC 1**	Few cilia	RGMC	MCIDAS homozygous (p.Cys147*)	Not tested	No cilia

PCD: primary ciliary dyskinesia; CBP: ciliary beat pattern; EM: electron microscopy; BEGM: bronchial epithelial growth medium; RGMC: reduced generation of multiple motile cilia; ALI: air–liquid interface; MTD-L-IDA: microtubules disorganisation lack of internal dynein arm. **^#^**: in two cases a single variant was found, but with a typical PCD diagnosis and ciliary defects that are recognised as consistent with being linked to the affected gene; **^¶^**: dyskinetic is defined as any abnormal ciliary beat pattern in healthy donors; **^+^**: twitch refers to markedly reduced ciliary beat amplitude; ^§^: viewed edge on; ^ƒ^: viewed from above.

### Co-cultured PCD basal cells form mucociliary epithelia at an ALI

**FIGURE 2:**
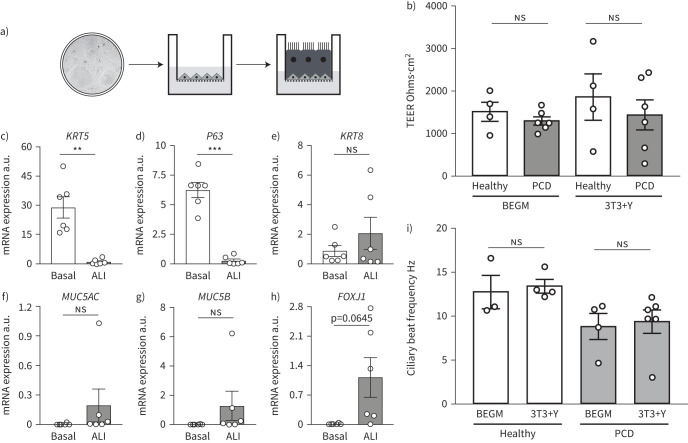
Ciliogenesis in cultured primary ciliary dyskinesia (PCD) epithelial cells *in vitro*. a) Schematic representation of air–liquid interface (ALI) culture in which basal cells are cultured to confluence and apical medium removed to stimulate ciliogenesis. The image in the two-dimensional culture is taken from figure 1b. b) Transepithelial electrical resistance (TEER) values in ALI cultures from basal cells expanded in bronchial epithelial growth medium (BEGM) or 3T3+Y from four healthy and six PCD donors after 28 days of culture. Each point represents a mean value of three consecutive readings from four wells for each individual. p-values were derived using a two-way ANOVA with Holm–Sidak's test for multiple comparisons, with statistical significance set at p<0.05. c–h) Quantitative PCR analysis compared the expression of keratin 5 (*KRT5*) (c), *P63* (d), *KRT8* (e), *MUC5AC* (f), *MUC5B* (g) and *FOXJ1* (h) between 3T3+Y basal cell cultures and the donor-matched differentiated ALI cultures after 28–30 days of culture. p-values were derived using two-tailed paired t-tests, with statistical significance set at p<0.05. i) Ciliary beat frequency analysis in ALI cultures from basal cells expanded in BEGM or 3T3+Y from three to six healthy and PCD donors for 28–35 days of ALI culture. We excluded PCD patient with static phenotypes from this analysis. p-values were derived using a two-way ANOVA with Holm–Sidak's test for multiple comparisons, with statistical significance set at p<0.05. ns: nonsignificant. **: p<0.01; ***: p<0.001.

Having established a method to extensively expand and cryopreserve nasal epithelial cells from PCD donors, we optimised methods to differentiate the cells at an ALI (figure 2a). This method allowed us to assess their ciliary phenotypes [8, 19, 28, 30]. PCD cells derived from 3T3+Y cultures formed confluent ALI cultures comparably to healthy controls. The barrier function of healthy and PCD-derived ALI cultures was also similar, as assessed by TEER measurements taken once cultures were fully differentiated (figure 2b). After 5 weeks of ALI culture, expression of the basal cell-associated genes *KRT5* (figure 2c) and *P63* (figure 2d) had markedly decreased and there were trends towards increased expression of the differentiation-associated genes *KRT8* (figure 2e), *MUC5AC* (figure 2f), *MUC5B* (figure 2g) and *FOXJ1* (a transcription factor expressed within the ciliated cell lineage; figure 2h), consistent with the expected differentiation of basal cell cultures towards mature epithelia containing ciliated cells and mucosecretory cells.

### PCD phenotypes are preserved in ALI cultures

**FIGURE 3:**
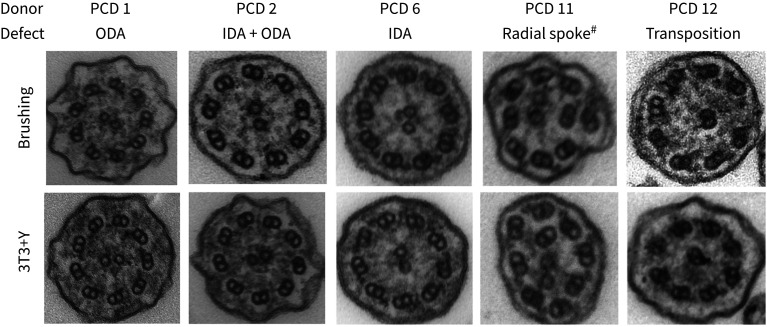
Persistent ultrastructural defects in primary ciliary dyskinesia (PCD) cell cultures. Electron micrographs of representative ciliary cross-sections illustrating examples of the defects observed in both initial patient brushings (upper panels) and in air­–liquid interface (ALI) cultures derived from 3T3+Y PCD primary epithelial cell cultures. For each patient, 3T3+Y-derived ALI cultures were assessed as follows: PCD 1 (219 cilia; 97.7% IDA, 2.3% no DA), PCD 2 (305 cilia; 100% DA), PCD 6 (231 cilia; 97.4% ODA, 2.6% no DA), PCD 11 (269 cilia; 98.1% ODA, 1.9% no DA; 13.6% microtubular defect) and PCD 12 (265 cilia; 98.1% normal, 0.4% ODA, 1.5% no DA; 9.3% transposed). ODA: outer dynein arm; IDA: inner dynein arm; DA: dynein arm. ^#^: radial spoke refers to axonemal disorganisation with absent IDAs.

A prerequisite for using cultured primary epithelial cells for diagnostic and basic research purposes is that ciliated cells generated in ALI cultures maintain their PCD phenotypes. Cilia were readily detectible using high-speed video microscopy in all cultures with the exception of RGMC1, where they were absent (as expected based on the patient phenotype). We found that ciliary beat frequency in ALI cultures from healthy control and PCD patients was consistent between basal cell expansion conditions (figure 2i). Cilia generated by healthy donor nasal epithelial cells retained a normal ciliary beat pattern in ALI culture (table 1), as expected based on prior experiments on bronchial epithelial cells [8]. Importantly, PCD patient ALI cultures maintained the ciliary beat defects that were present in the original nasal brushings, after basal cell culture in either BEGM or 3T3+Y conditions and subsequent differentiation. PCD patient cells showed completely static, stiff or dyskinetic cilia that were consistent with the original nasal brushings and that reflected the ciliary beat patterns expected for the respective underlying genetic defects. On measuring the beat frequency (figure 2i) and beat pattern, very similar results were observed between the direct patient biopsies and the cultured cells (table 1). The expected ultrastructural defects were also recapitulated in the cell cultures (figure 3). In these experiments, 3T3+Y culture allowed us to analyse ciliary beat frequency and pattern for one healthy donor and three PCD donors for whom failed cultures would have prevented this analysis using traditional BEGM culture (figure 2i, table 1).

### Miniaturisation of PCD ALI cultures

**FIGURE 4:**
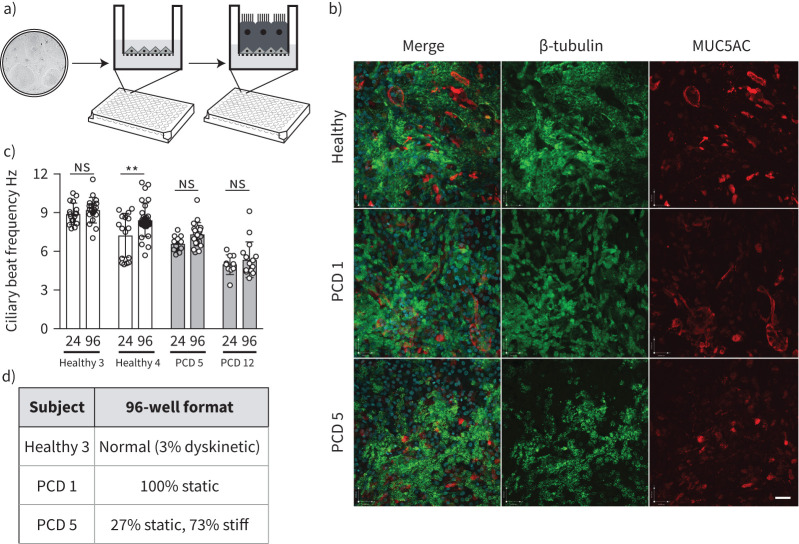
Miniaturisation of air–liquid interface (ALI) cultures to 96-well format to generate primary ciliary dyskinesia (PCD) patient models. a) Schematic showing experimental set-up for immunofluorescence screening in 96-well transwell ALI system. The image in the two-dimensional culture is taken from figure 1b. b) Immunofluorescence images demonstrating the presence of β-tubulin-expressing cilia (green) and MUC5AC-expressing mucosecretory cells (red) in 96-well format ALI cultures. Two PCD donors, one with static cilia (PCD 1) and one with circular cilia (PCD 5) defects, are shown in comparison to a healthy donor control. Images are representative of two healthy donors and four PCD donors tested. Scale bar: 50 µm. c) Ciliary beat frequency analysis comparing multiple recordings from two healthy donors (Healthy 3 and 4) and two PCD patients (PCD 5 and 12) in 24-well and 96-well formats. Data points are individual recordings from four to five wells, with five videos recorded per well. p-values were derived using a two-way ANOVA with Holm–Sidak's test for multiple comparisons, with statistical significance set at p<0.05. ns: nonsignificant. **: p<0.01. d) Ciliary beat pattern analysis in 96-well format. 24-well equivalent values are found in table 1.

The ability to expand patient-specific primary PCD epithelial cells with high success rates and assess their ciliary function in culture represents an advance over traditional culture methods for human nasal epithelial cells. With the knowledge that we could provide large numbers of basal cells as the input from individual patients, we next sought to miniaturise PCD ALI cultures to a 96-transwell format that would be compatible with compound screening using microscopy (figure 4a). We assessed the ability of cultures from five donors to differentiate in this format, comprising two healthy donors (Healthy 3 and 4; table 1), two PCD patients with a circular beat pattern (PCD 5 and 12; table 1) and one PCD patient with static cilia (PCD 1; table 1). All donor cultures formed a confluent epithelium containing both MUC5AC^+^ mucosecretory cells and β-tubulin^+^ ciliated cells (figure 4b), which could be readily visualised using high-speed video microscopy (supplementary video). Cultures were visually inspected for ciliation and the proportion of the surface that was ciliated appeared consistent between 24-well and 96-well formats. Analysis of ciliary beat frequency (figure 4c) and pattern (figure 4d) showed consistent findings between the 96-well and the 24-well format used above. While a small, statistically significant increase in beat frequency was seen in one healthy donor in the 96-well format (figure 4c), this is unlikely to be biologically significant because all data points remained within the expected normal range.

### Readthrough agents in combination with NMD inhibitors increase basal body formation

**FIGURE 5:**
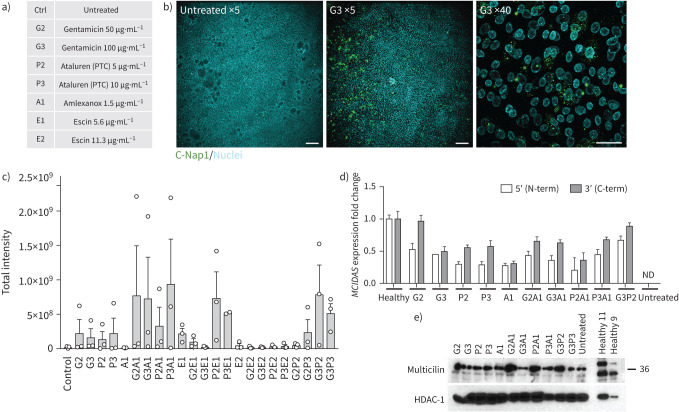
Immunofluorescence screening to assess readthrough therapies in a patient with *MCIDAS-*mutated reduced generation of multiple motile cilia (RGMC) ciliopathy. a) Key for drug names and doses for immunofluorescence screening in patient RGMC 1. b) An example of basal body formation in the presence of 100 µg·mL^−1^ gentamicin (G3) readthrough drug treatment shows the immunofluorescence approach based on co-staining of C-Nap1 (a basal body-associated protein; green, fluorescein isothiocyanate) and Hoechst 33258 counterstain (cyan, 4′,6-diamidino-2-phenylindole). Scale bars: 200 μm (×5 images) and 34 μm (×40 image). Examples from each condition are shown in supplementary figure S3. c) Quantification of the formation of basal bodies after treatment with the combinations of readthrough drugs and nonsense-mediated decay inhibitors shown in (a), based on total intensity of C-Nap1 staining, quantified using a custom-made ImageJ macro. An untreated patient control was compared to treated patient samples. No significant differences were found in a one-way ANOVA with Holm–Sidak's test for multiple comparisons. d) Quantitative PCR analysis comparing *MCIDAS* mRNA abundance between drug-treated conditions on the patient cells *versus* a healthy control donor in independent cultures using conventional 12-well transwell supports after 7 days of air–liquid interface (ALI) culture. No mRNA expression was detected in untreated cultures. e) Western blot analysis of multicilin expression in drug-treated conditions in independent cultures using conventional 12-well transwell supports at day 7 post-ALI. MCIDAS: multiciliate differentiation and DNA synthesis associated cell cycle; ND: not detected; HDAC-1: histone deacetylase 1.

Because gentamicin is toxic in humans at serum concentration ranges that allow readthrough in cell cultures [31] and long-term use of nebulised gentamicin may increase systemic toxicity, we assessed readthrough drugs (gentamicin and ataluren) in combination with NMD inhibitors (amlexanox, escin) in an attempt to improve the therapeutic relevance of our findings. In 96-well format, we treated nasal epithelial cells from a patient with an RGMC ciliopathy, which is caused by a homozygous nonsense mutation (c.441C>A; p.Cys147*) in the *MCIDAS* gene, with dose ranges of gentamicin (50–100 μg·mL^−1^) and ataluren (2.5–10 μg·mL^−1^) in combination with NMD inhibitors (figure 5a). Drugs were included from the point of ALI creation onwards, because multicilin expression precedes ciliogenesis (supplementary figure S2) [19]. After 12 days, we assessed expression of C-Nap1, a centriolar protein that co-localises with β-tubulin^+^ cilia in cultured nasal epithelia [32, 33], using an Opera Phenix high-content screening confocal microscopy system. C-Nap1 expression in healthy control cells showed co-localisation with β-tubulin at day 12 post-ALI (supplementary figure S3). In untreated control conditions, the epithelium contained few C-Nap1^+^ basal bodies (figure 5b, c, supplementary figures S1 and S4). However, increased basal body formation was seen across various combinations of drug treatments (figure 5c, supplementary figure S1). This was more clearly evident using higher resolution confocal microscopy because individual C-Nap1^+^ cells were visible (figure 5b). MCIDAS protein was not detected after day 10 in ALI cultures (supplementary figure S5) and *MCIDAS* mRNA was undetectable by qPCR in untreated cultures at day 7 post-ALI, but was restored to varying extents after drug treatment was repeated in independent 12-well format ALI cultures (figure 5d). Despite this, in longer-term experiments using the most promising drug combinations, we were not able to observe the formation of cilia by either video microscopy or immunofluorescence staining after 28 days of culture (data not shown).

**FIGURE 6:**
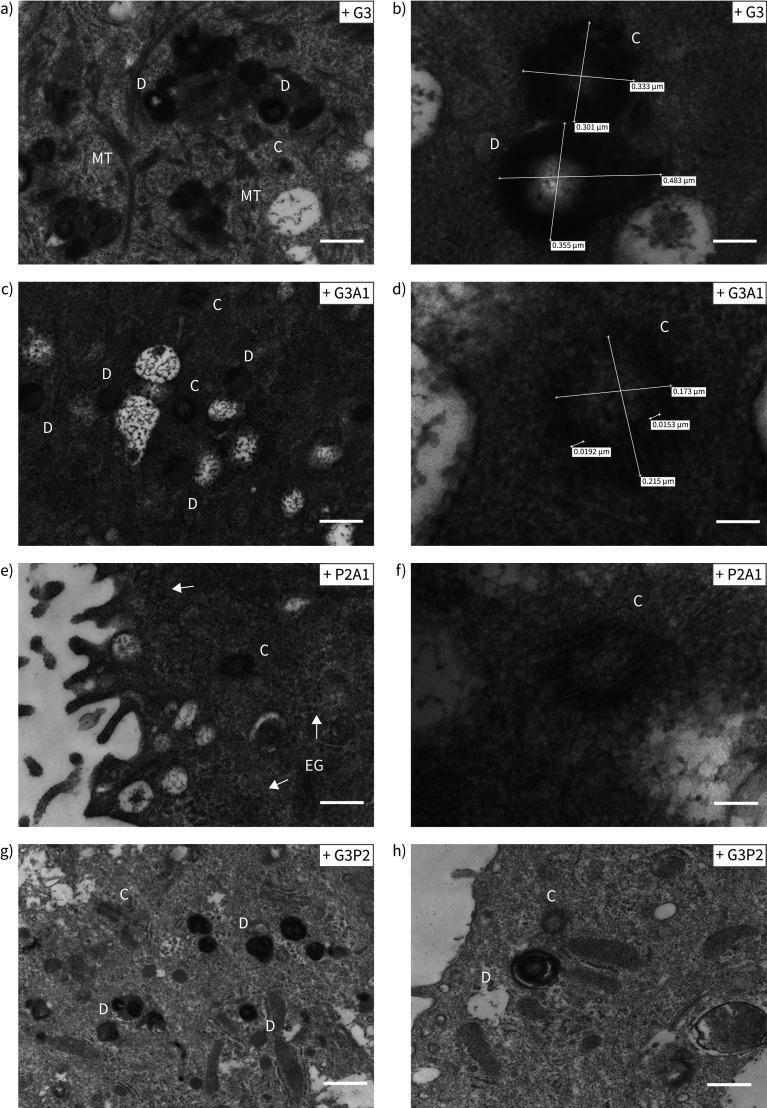
High magnification images of the structures found in drug-treated reduced generation of multiple motile cilia (RGMC) cells. The different panels show representative high magnification images of accumulation of basal bodies “precursors” observed in RGMC cells after treatment with a, b) G3 (scale bar: 400 nm in a, 100 nm in b); c, d) G3A1 (scale bar: 400 nm in c, 50 nm in d); e, f) P2A1 (scale bar: 400 nm in e, 100 nm in f); and g, h) G3P2 (scale bar: 800 nm in g, 400 nm in h). Electron-dense deuterosomes were observed in high number and we observed several centrioles showing a “cartwheel” structure. Centriole size was ∼0.2 μm diameter (c–f [50]). None of these structures were observed in untreated controls. In (e), arrows indicate electron-dense granules. D: deuterosomes; C: centrioles; MT: microtubules agglomeration; EG: electron-dense granules.

Recovery of full-length multicilin protein was assessed by Western blot in these cultures; increased expression of a band at the expected 38 kDa size was detected in multiple drug treatment conditions compared to control untreated patient cell cultures and healthy control cultures (figure 5e). TEM showed the formation of basal bodies of a range of maturities (figure 6, supplementary figure S6). Basal body biogenesis is driven by different mechanisms, with the canonical mechanism involving duplication of pre-existing centrioles. However, formation of multiple basal bodies in multiciliated cells can also follow a non-canonical pathway that is mediated by the formation of structures called deuterosomes [34]. Just prior to deuterosome formation, electron-dense “fibrogranular material” enriched in microtubules is observed [34]. We detected this type of microtubular agglomeration in RGMC cells treated with readthrough agents (figure 6). These electron-dense granules then condense to form hollow, spherical structures called deuterosomes, which are also highly electron-dense [34]. Immature centrioles are amplified from daughter centrioles through deuterosome formation [35, 36]. We observed a number of what we believe might be deuterosomes in treated RGMC cells (figure 6, supplementary figure S6). Despite the apparent presence of deuterosomes, we did not observe procentrioles originating from them, although some structured centrioles could be found in the vicinity of deuterosome-like structures. These centrioles had the characteristic “cartwheel” structure of immature centrioles and were ∼0.2 μm in diameter (figure 6), as reported previously [37].

## Discussion

Here we show that, in conditions that allow robust epithelial cell expansion, PCD nasal epithelial cells express proteins characteristic of basal cells, form differentiated mucociliary epithelia in ALI cultures and retain patient-specific ciliary dyskinesia phenotypes as previously described for low passage cultures in BEGM [28, 29]. The consistent culture initiation, differentiation capability after extensive culture, absence of viral immortalisation [38] and reliable cryopreservation [39] afforded by this approach are a major benefit for PCD research. This, along with other advances in primary airway epithelial cell culture [40–42], will encourage the development of large biobanks of well-phenotyped patient-specific PCD epithelial cells of known genotype and will allow repeated experimentation in the same genetic background.

Current therapeutic avenues in PCD focus upon early diagnosis and clinical management in order to prevent lung function decline. However, at present, no therapies that restore ciliary function are available. In the future, the ability to expand significant numbers of human epithelial cells may also open the door to gene editing, mRNA, oligonucleotide and cell therapy approaches in PCD. Notably, cell therapies using epithelial cells expanded on 3T3-J2 feeder layers are approved for clinical use for severe burns and limbal stem cell deficiency [43–45] and expansion from single cell-derived clones is achievable [46]. However, because cellular therapy in genetic lung disease remains a distant prospect [47], model systems that enable the testing of gene therapies or small molecule approaches are much needed. To this end, the production of many cells from individual donors allowed us to miniaturise ALI cultures to 96-well format. This in turn allowed personalised investigation using higher throughput, immunofluorescence-based compound screening in the context of a case of RGMC. In a patient with an *MCIDAS* mutation, we were able to demonstrate, by immunofluorescence screening of the basal body protein C-Nap1, that combined readthrough and NMD inhibition enhanced basal body formation, although the cultures did not produce motile cilia. This might have been due to the limited range of concentrations that we could test in differentiated cultures: at higher doses, compounds like gentamicin, amlexanox and escin are toxic. Another possibility is that the readthrough transcript generated a miss-folded protein unable to generate the protein–protein interactions and complex three-dimensional structures required for the generation of functional cilia. We further demonstrate the feasibility of screening 96-well format cultures using high-speed video microscopy, meaning that this approach could also be used to screen for agents that correct ciliary beat pattern defects seen in other PCD phenotypes.

Cultures containing only basal epithelial cells can have limitations for respiratory disease modelling owing to the lack of barrier function, the absence of differentiated cell types [48] and the preferential replication of some viruses in specific epithelial cell types (*e.g.* respiratory syncytial virus in multiciliated cells [49]). ALI culture is typically low-throughput and thus not well suited to screening applications. Although drugs were applied basolaterally in cell culture medium, it is possible to develop a system in which aerosolised drugs are applied to cultures. As such, the approach described here is likely to be widely applicable in basic lung biology, in inhalation toxicology [50] and for investigations of other respiratory diseases with epithelial involvement, such as chronic obstructive pulmonary disease, asthma and pulmonary fibrosis. Organoid culture has been developed as a scalable primary cell culture model in the context of cystic fibrosis [51], in which organoid swelling can be used as a proxy for ion channel function [52, 53]. However, this approach, even with the development of airway organoids [54, 55], lends itself less well to ciliary diseases because ciliary motility is harder to visualise within the organoid structure than in ALI cultures and the application of therapeutic compounds to the apical surface also poses a technical challenge.

Our approach enables the generation and long-term maintenance of differentiation-competent primary epithelial cell cultures from PCD patients with diverse causative mutations. As a result, it is relevant for the diagnosis of PCD and for basic PCD research, and to our knowledge is the first example of a personalised screening approach in a rare respiratory disease using differentiated human respiratory epithelial cells. The culture method and assay miniaturisation suggest readthrough drugs and NMD inhibitors as targets for further pre-clinical exploration in the subset of PCD patients with nonsense mutations.

## Shareable PDF

10.1183/13993003.00455-2020.Shareable1 This one-page PDF can be shared freely online.
Shareable PDF ERJ-00455-2020.Shareable
