# Supplementary material for: Higher throughput drug screening for rare respiratory diseases: readthrough therapy in primary ciliary dyskinesia
Source: Eur Respir J. 2021 Oct 14;58(4):2000455. doi: 10.1183/13993003.00455-2020 (PMC8514977; doi:10.1183/13993003.00455-2020)
Supplement: Supplementary file 1 [file ERJ-00455-2020.Shareable.pdf]

EUROPEAN RESPIRATORY JOURNAL
ORIGINAL RESEARCH ARTICLE
D.D.H. LEE ET AL.

SHAREABLE PDF

Higher throughput drug screening for rare respiratory diseases: readthrough therapy in primary ciliary dyskinesia

Dani Do Hyang Lee [1,11], Daniela Cardinale[1,11], Ersilia Nigro [2], Colin R. Butler [2], Andrew Rutman[3], Mahmoud R. Fassad [4,5], Robert A. Hirst [3], Dale Moulding [6], Alexander Agrotis [7], Elisabeth Forsythe[4], Daniel Peckham [8], Evie Robson[8], Claire M. Smith [1], Satyanarayana Somavarapu [9], Philip L. Beales[4], Stephen L. Hart[4], Sam M. Janes [2], Hannah M. Mitchison [4], Robin Ketteler [7], Robert E. Hynds [2,10,12] and Christopher O'Callaghan[1,3,12]

[1]UCL Great Ormond Street Institute of Child Health, London, UK. [2]Lungs for Living Research Centre, UCL Respiratory, Division of Medicine, University College London, London, UK. [3]Centre for PCD Diagnosis and Research, Dept of Respiratory Sciences, University of Leicester, Leicester, UK. [4]Ciliary Disease Section, Genetics and Genomic Medicine Research and Teaching Dept, UCL Great Ormond Street Institute of Child Health, London, UK. [5]Dept of Human Genetics, Medical Research Institute, Alexandria University, Alexandria, Egypt. [6]Developmental Biology and Cancer, UCL Great Ormond Street Institute of Child Health, London, UK. [7]MRC Laboratory for Molecular Cell Biology, University College London, London, UK. [8]Leeds Institute for Medical Research, University of Leeds, Leeds, UK. [9]Dept of Pharmaceutics, UCL School of Pharmacy, University College London, London, UK. [10]UCL Cancer Institute, University College London, London, UK. [11]D.D.H. Lee and D. Cardinale contributed equally. [12]R.E. Hynds and C. O'Callaghan contributed equally to this article as lead authors and supervised the work.

Corresponding author: Christopher O'Callaghan (cocallaghan@ucl.ac.uk)

Check for updates

Shareable abstract (@ERSpublications)
Primary cell culture of nasal epithelial cells (including differentiation to multiciliated cells) from patients with primary ciliary dyskinesia enabled immunofluorescence-based screening in miniaturised air–liquid interface cultures https://bit.ly/3rjoxBF

Cite this article as: Lee DDH, Cardinale D, Nigro E, *et al*. Higher throughput drug screening for rare respiratory diseases: readthrough therapy in primary ciliary dyskinesia. *Eur Respir J* 2021; 58: 2000455 [DOI: 10.1183/13993003.00455-2020].

This single-page version can be shared freely online.

Copyright ©The authors 2021.

This version is distributed under the terms of the Creative Commons Attribution Licence 4.0.

This article has supplementary material available from erj.ersjournals.com

Received: 27 Feb 2020
Accepted: 1 March 2021

Abstract

Background Development of therapeutic approaches for rare respiratory diseases is hampered by the lack of systems that allow medium-to-high-throughput screening of fully differentiated respiratory epithelium from affected patients. This is a particular problem for primary ciliary dyskinesia (PCD), a rare genetic disease caused by mutations in genes that adversely affect ciliary movement and consequently mucociliary transport. Primary cell culture of basal epithelial cells from nasal brush biopsies followed by ciliated differentiation at the air–liquid interface (ALI) has proven to be a useful tool in PCD diagnostics but the technique's broader utility, including in pre-clinical PCD research, has been restricted by the limited number of basal cells that can be expanded from such biopsies.

Methods We describe an immunofluorescence screening method, enabled by extensive expansion of basal cells from PCD patients and the directed differentiation of these cells into ciliated epithelium in miniaturised 96-well transwell format ALI cultures. As proof-of-principle, we performed a personalised investigation in a patient with a rare and severe form of PCD (reduced generation of motile cilia), in this case caused by a homozygous nonsense mutation in the *MCIDAS* gene.

Results Initial analyses of ciliary ultrastructure, beat pattern and beat frequency in the 96-well transwell format ALI cultures indicate that a range of different PCD defects can be retained in these cultures. The screening system in our proof-of-principal investigation allowed drugs that induce translational readthrough to be evaluated alone or in combination with nonsense-mediated decay inhibitors. We observed restoration of basal body formation but not the generation of cilia in the patient's nasal epithelial cells *in vitro*.

Conclusion Our study provides a platform for higher throughput analyses of airway epithelia that is applicable in a range of settings and suggests novel avenues for drug evaluation and development in PCD caused by nonsense mutations.
